# Probing the Structure and Function of the Cytosolic Domain of the Human Zinc Transporter ZnT8 with Nickel(II) Ions

**DOI:** 10.3390/ijms22062940

**Published:** 2021-03-14

**Authors:** Maria Carmen Catapano, Douglas S. Parsons, Radosław Kotuniak, Přemysl Mladěnka, Wojciech Bal, Wolfgang Maret

**Affiliations:** 1 Departments of Biochemistry and Nutritional Sciences, School of Life Course Sciences, Faculty of Life Sciences and Medicine, King’s College London, Franklin-Wilkins Bldg, 150 Stamford St., London SE1 9NH, UK; catapanm@faf.cuni.cz (M.C.C.); parsonsd@bu.edu (D.S.P.); 2 Department of Analytical Chemistry, Faculty of Pharmacy in Hradec Králové, Charles University, Heyrovského 1203, 500 05 Hradec Králové, Czech Republic; 3 Department of Radiology, Boston University School of Medicine, 670 Albany Street, Boston, MA 02118, USA; 4 Institute of Biochemistry and Biophysics, Polish Academy of Sciences, Pawińskiego 5a, 02-106 Warsaw, Poland; r.kotuniak@ibb.waw.pl (R.K.); wojciech.bal.ibb@gmail.com (W.B.); 5 Department of Pharmacology and Toxicology, Faculty of Pharmacy in Hradec Králové, Charles University, Heyrovského 1203, 500 05 Hradec Králové, Czech Republic; mladenkap@faf.cuni.cz

**Keywords:** zinc transporter, ZnT8, C-terminal domain, zinc, nickel, diabetes type 2

## Abstract

The human zinc transporter ZnT8 provides the granules of pancreatic β-cells with zinc (II) ions for assembly of insulin hexamers for storage. Until recently, the structure and function of human ZnTs have been modelled on the basis of the 3D structures of bacterial zinc exporters, which form homodimers with each monomer having six transmembrane α-helices harbouring the zinc transport site and a cytosolic domain with an α,β structure and additional zinc-binding sites. However, there are important differences in function as the bacterial proteins export an excess of zinc ions from the bacterial cytoplasm, whereas ZnT8 exports zinc ions into subcellular vesicles when there is no apparent excess of cytosolic zinc ions. Indeed, recent structural investigations of human ZnT8 show differences in metal binding in the cytosolic domain when compared to the bacterial proteins. Two common variants, one with tryptophan (W) and the other with arginine (R) at position 325, have generated considerable interest as the R-variant is associated with a higher risk of developing type 2 diabetes. Since the mutation is at the apex of the cytosolic domain facing towards the cytosol, it is not clear how it can affect zinc transport through the transmembrane domain. We expressed the cytosolic domain of both variants of human ZnT8 and have begun structural and functional studies. We found that (i) the metal binding of the human protein is different from that of the bacterial proteins, (ii) the human protein has a C-terminal extension with three cysteine residues that bind a zinc(II) ion, and (iii) there are small differences in stability between the two variants. In this investigation, we employed nickel(II) ions as a probe for the spectroscopically silent Zn(II) ions and utilised colorimetric and fluorimetric indicators for Ni(II) ions to investigate metal binding. We established Ni(II) coordination to the C-terminal cysteines and found differences in metal affinity and coordination in the two ZnT8 variants. These structural differences are thought to be critical for the functional differences regarding the diabetes risk. Further insight into the assembly of the metal centres in the cytosolic domain was gained from potentiometric investigations of zinc binding to synthetic peptides corresponding to N-terminal and C-terminal sequences of ZnT8 bearing the metal-coordinating ligands. Our work suggests the involvement of the C-terminal cysteines, which are part of the cytosolic domain, in a metal chelation and/or acquisition mechanism and, as now supported by the high-resolution structural work, provides the first example of metal-thiolate coordination chemistry in zinc transporters.

## 1. Introduction

A subgroup of four out of ten human zinc transporters (ZnT2, 3, 4 and 8) of the cation diffusion facilitator (CDF) protein family exports Zn^2+^ ions from the cytosol into intracellular vesicles [1,2]. One of the four, ZnT8 (*SLC30A8*), is highly expressed in the membrane of the dense insulin secretory granules of pancreatic β-cells, where it supplies zinc(II) ions for the storage of insulin as a crystalline hexamer and for other aspects of granule biochemistry [3]. ZnT8 is also present in α-cells of the endocrine pancreas, but its precise role in glucagon secretion has not yet been elucidated [4].

Concepts of how these vesicular zinc transporters work were primarily based on the 3D structures of prokaryotic CDF proteins. The crystal structure of the *Escherichia coli* protein YiiP revealed a homodimer with a transmembrane domain (TMD) and a cytosolic C-terminal domain (CTD) [5]. The dimer binds eight Zn(II) ions: two in sites A, which are the primary TMD transport sites, two in sites B between the domains and with an unknown function, and four in the binuclear sites C at the dimer interface of the CTDs. The CTDs in *E. coli* YiiP are believed to be metal ion sensors, working by an allosteric mechanism such that the occupancy of sites C with zinc induces a conformational change of the CTD, which is then transmitted to the TMD for triggering zinc/proton antiport [6]. A cryogenic electron microscopy structure of a YiiP homologue from *Shewanella oneidensis*, however, does not confirm the allosteric mechanism of zinc binding, but rather suggests that Zn(II) ions are bound to the CTD with such high affinity as to be considered structurally stabilizing for the dimer as a whole [7]. Aside from YiiP, there are currently crystal structures of bacterial CTDs from *Thermus thermophilus* CzrB [8], *Thermotoga maritima* TM0876 [9], *Magnetospira* sp. MamB [10], *Magnetospirillum gryphiswaldense* MamM [11], and CzcD proteins from *T. thermophilus*, *Pseudomonas aeruginosa* and *Cupriavidus metallidurans* [12]. The structural models of the bacterial proteins fail to explain the function(s) of the CTD in the mammalian family of vesicular transporters. While the bacterial proteins sense and export an excess of zinc, there is no evidence for an excess of zinc in the cytosol of eukaryotic cells for export into granules of the secretory pathway. While total cellular zinc concentrations are about 250 µM, the Zn(II) ion concentration available to fluorescent probes and sensors is only hundreds of pM to maximally 1.5 nM [13,14]. In granules, estimates of Zn^2+^ ion concentrations are 120 nM (pH 6) and total Zn(II) concentrations are tens to perhaps even hundreds of mM [15,16]. The Zn(II) ion concentration thus measured have been variously referred to accessible, buffered, labile, available or “free” zinc with each of these attributes having limitations and/or being misleading in the implied meaning. Given the abundance of low molecular weight ligands in the cell, there are no Zn^2+^ ions with water ligands only in the cytosol, but several nM of zinc bound to small molecules with an overall approximate affinity of 100 fM [17]. Independent of these fundamental issues, there is no excess of zinc freely available for passive transmembrane transport. Additional issues for interpreting gradients across the membrane of granules are that given an average granule volume of only 7.2 aL, the estimated Zn^2+^ concentration of 120 nM would indicate less than one Zn^2+^ ion per granule, and that crystallization of insulin will further reduce the concentration in solution as it removes zinc from equilibria in solution [18].

It is also the case that the protective effects of bacterial export proteins may not require a constitutively active protein, and so an allosteric mechanism relying on a toxic excess of cytosolic zinc fulfils the necessary function in bacteria. Conversely, mammalian vesicular ZnTs must supply their secretory granules with zinc under ‘normal’ tightly controlled cytosolic zinc, and thus a simple allosteric mechanism reliant on increased cytosolic zinc may not be possible.

Two cryo-EM investigations of human ZnT8, one at relatively low resolution (about 20 Å) and another at relatively high resolution (about 4 Å), have now provided additional insights [19,20] and confirm the differences we noted in zinc binding of the CTD compared to the bacterial YiiP protein and the involvement of the C-terminal cysteines in zinc binding [21]. The metal sensing mechanism of the CTD discussed for the YiiP protein is based on the presence of two pairs of binuclear metal ion sites at the dimer interface. Each Zn(II) ion is bound by a pair of two His residues and a bridging Asp side chain from the second subunit. A trinuclear site C has been observed in the bacterial CzrB protein [8] and one, two, or three metal binding sites have been observed in the CzcD proteins [12]. Our analysis showed that in the human vesicular ZnTs, the ligands for binding of the second metal ion, including the bridging Asp, are not strictly conserved [21]. The conservation of only three potential ligands in the vesicular zinc transporters raises the questions of whether the CTDs in these transporters sense Zn(II) concentrations and how the monomers interact in the dimer. Related to the nature of the interface metal site is the question of how zinc is delivered to and from the transporter as cellular zinc traffic is tightly regulated and all Zn(II) ions are bound to biological ligands [17]. The protein fold of the CTD resembles that of copper metallochaperones [5]. Thus, does the CTD have the function of a chaperone for delivery of zinc or is it interacting with a putative metallochaperone to acquire zinc? Rather than solving these questions, the remarkably different zinc binding of the CTD of human ZnT8 involving both the N-terminus of the transmembrane domain and the C-terminus of the CTD puts new emphasis on their future resolution [20].

A structural issue of ZnT8 with significant functional implications is a point mutation at position 325 in the CTD. It results in two variants with either Arg (R) or Trp (W), both of which are common in human populations (R: 60–95%; W: 5–40%) [22]. Remarkably, the major R-variant increases the risk of developing type 2 diabetes [3]. The transport activity of the R-variant was found to be higher than that of the W-variant [23], lower [24] or no difference was observed [25], leaving the question unanswered as to whether less or more zinc in the insulin granules is favourable to β-cell health. A computational investigation has now shown that the two amino acids differentially affect zinc coordination sites in the TMD [26]. They affect the orientation of the transmembrane (TM) helices 4 and 5, which increases the distances for the zinc ligands (Asp)D110 and D224 in site A in the W-variant. Furthermore, (Glu)E88, moves closer to site A. E88 together with D103 are thought to serve as ligands of the zinc(II) ion once it moves away from the two histidine ligands (H106 and H220) in site A. The two ZnT8 variants are targeted by different autoantibodies in type 1 diabetes, indicating that the mutation also affects epitope formation, although there is no apparent change in type 1 diabetes risk [27]. Thus, characterizing the CTDs of ZnT8 functionally in terms of metal binding would solve a key issue in β-cell granule biology, provide important information regarding the biology of other zinc-containing vesicles served by ZnT2-4, and make a significant contribution to CDF biology in general with wide implications.

In this work, we expressed both common CTD variants of ZnT8 and employed their nickel binding as spectroscopic probes for their structures. We describe differences in metal affinities and coordination of the two variants and establish for the first time an important structural difference between the variants. We also further characterized metal binding to the ZnT8 C-terminal cysteines using both recombinant CTD protein and a synthetic peptide of the C-terminus. The low nanomolar affinity of the peptide for Zn(II) suggests that the C-terminus, but not the N-terminus, which binds zinc with an affinity lower by two orders of magnitude, could be involved in a “swinging arm mechanism” in which the C-terminus in the extended conformation acquires zinc from a zinc donor and then swings over to form the metal site with the other ligands for allosteric regulation of zinc transport. In agreement with the recent 3D structural work on human ZnT8, our investigations confirm the novel involvement of metal-sulfur interactions in the biochemistry of zinc transporter proteins.

## 2. Results

### 2.1. Stability of the ZnT8 CTDs

**Figure 1:**
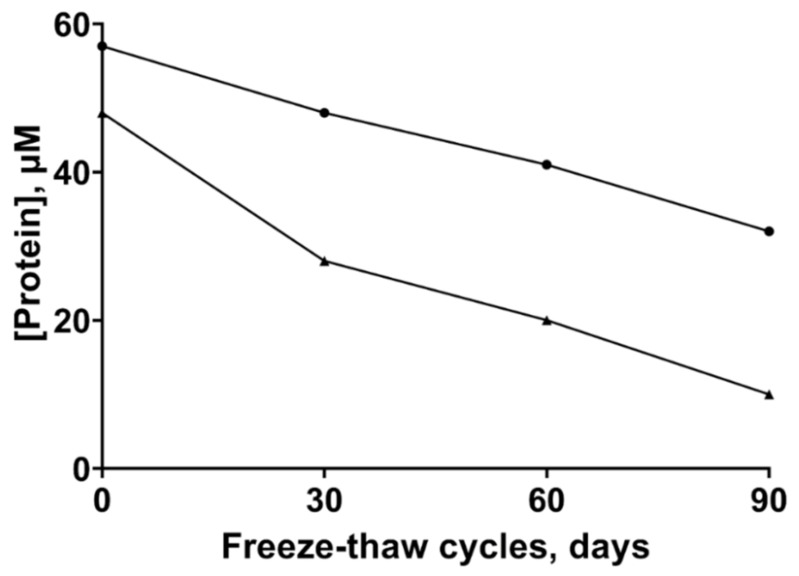
ZnT8c protein stability. Both ZnT8cR (circles) and ZnT8cW (triangles) were stored at −20 °C in 50 mM TRIS/HCl, pH 8, 500 mM NaCl, 300 mM imidazole, 2 mM DTT (dithiothreitol), 100 mM sucrose with 50% (*v*/*v*) glycerol. After each of three freeze–thaw cycles the concentration of soluble protein was determined spectrophotometrically.

Since we noticed a propensity of the ZnT8c proteins to aggregate within days of purification [21], we examined ways to store the protein. The protein was stored at −20 °C with a solution containing 50% (*v*/*v*) glycerol for up to three months, subjected to several freeze–thaw cycles and then used immediately after thawing. Following thawing, the protein was briefly and lightly vortex-mixed and centrifuged at 14,000× *g* for 5 min. The supernatant was collected, and the soluble protein concentration determined spectrophotometrically. After three freeze–thaw cycles over three months the soluble protein concentration decreased from 48 to 10 µM for ZnT8cW and from 57 to 32 µM for ZnT8cR (Figure 1). Unless the slightly different initial protein concentration has a strong stabilizing effect, the loss of soluble protein (79 vs. 44%) indicates that the ZnT8cR variant is more stable, consistent with the higher thermostability of this variant [21].

### 2.2. Electronic Absorption Spectra

**Figure 2:**
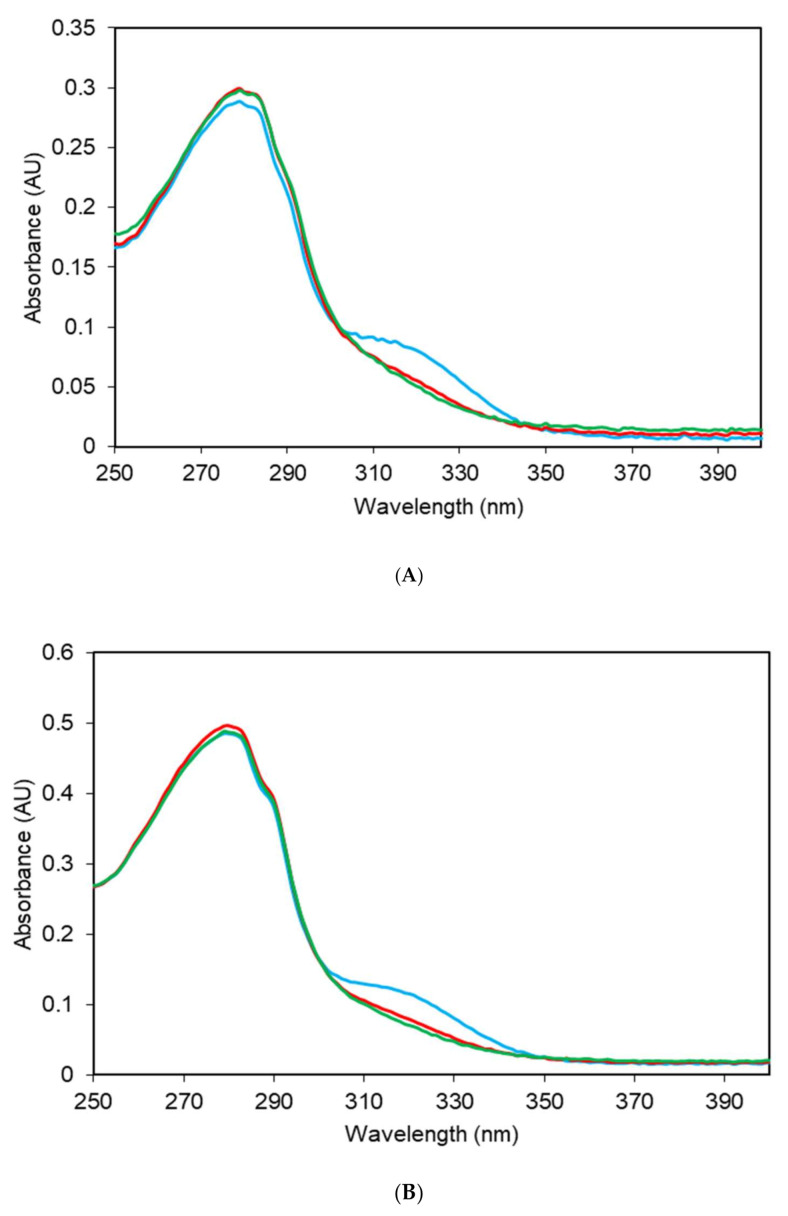
ZnT8c UV absorbance changes with addition of zinc. Representative (n = 3) UV absorbance spectra of 35 µM of ZnT8cR (**A**) and ZnT8cW (**B**) in the absence of Zn^2+^ (blue), and with one (red) and two (green) molar equivalents of Zn^2+^, demonstrating that addition of Zn^2+^ ablates a broad peak at approximately 320 nm from both apo-ZnT8c variants. Spectra are buffer- and dilution-corrected.

The UV absorption spectra of apo-ZnT8cR or apo-ZnT8cW—defined as no added metal following purification [21]—showed a broad peak at approximately 320 nm, in addition to the typical peaks contributed by aromatic residues at 275–290 nm (Figure 2). The absorbance at 320 nm indicates bound nickel. Addition of up to two molar equivalents of Ni^2+^ to the apo-proteins did not affect the peak at 320 nm. However, upon addition of ZnCl_2_, the peak at 320 nm was lost (Figure 2), indicating that the spectroscopically silent Zn^2+^ displaces the Ni^2+^.

### 2.3. Inductively Coupled Plasma-Mass Spectrometry (ICP-MS) and Total Reflection X-ray Fluorescence (TXRF) Metal Analyses

The unexpected presence of nickel bound to both ZnT8c variants as indicated by the UV absorbance led us to perform further metal analyses of the proteins. Both apo-variants were purified as previously [21], diluted to 10 µM and incubated with 0–10 molar equivalents of ZnCl_2_. Following gel filtration to remove excess Zn^2+^, the zinc and nickel content of the proteins was measured using ICP-MS (Table 1 and Table 2). Following gel filtration, the protein concentration of both variants was 2 μM as measured spectrophotometrically. The two apo-ZnT8c variants, with no additional metal added, contained 0.15–0.19 total Zn^2+^ and Ni^2+^ ions per monomer (n = 3; total metal content in Table 1 and Table 2 divided by 2 μM protein). Over 80% of this residually bound metal was contributed by Ni^2+^. Addition of an excess of Zn^2+^ displaced the bound nickel and the zinc binding capacity was three zinc ions per monomer for both ZnT8c variants, in agreement with our previous zinc stoichiometry data [21].

To determine the reason for the higher-than-expected amount of residual nickel bound to the proteins, the source of nickel in the initial preparation was traced to the purification buffers. The buffer preparation protocol was subsequently altered and routine checks performed to control the residual nickel content. Following these changes, total reflection X-ray fluorescence TXRF analyses were performed to determine the protein Ni and Zn content (Table 3). We then compared the results obtained by the two different methods of analysis by calculating the metal stoichiometry of the ZnT8c proteins following the change in buffer preparation (with no metal added to the protein solutions during or following purification). Using inductively coupled plasma-mass spectrometry (ICP-MS) analysis, the calculated stoichiometries (metal:protein per monomer) were 0.5 ± 0.05:1 nickel and 0.1 ± 0.06:1 zinc per monomer for ZnT8cW; and 0.6 ± 0.1:1 nickel and 0.2 ± 0.1:1 zinc for ZnT8cR. TXRF analysis indicated that the stoichiometries for ZnT8cW were 0.2 ± 0.1:1 nickel and 0.1 ± 0.1:1 zinc per monomer, while ZnT8cR contained 0.3 ± 0.1:1 nickel and 0.2 ± 0.1:1 zinc per monomer (Table 3).

The results for zinc were the same with both methods. ICP-MS showed a slightly higher nickel content of the proteins compared to the TXRF analysis. Importantly, the R-variant showed a consistently higher metal concentration, indicating a slightly higher affinity for both metal ions. After establishing the intrinsic metal stoichiometries of the purified proteins, the next issue to be addressed was the binding stoichiometry when nickel ions are added. The stoichiometry was previously established for zinc and confirmed here in Table 1 and Table 2 when also accounting for the nickel initially present in the proteins.

### 2.4. Thiol Assay

Since our previous experiments implicated the involvement of the cysteines at the C-terminus in metal binding, we employed the 5,5′-dithio-bis-(2-nitrobenzoic acid) (DTNB) assay to quantify the free sulfhydryls of both ZnT8c proteins [21]. Alkylation of the sulfhydryls with iodoacetamide was confirmed by measuring only 0.2–0.3 free sulfhydryls per monomer in the protein with DTNB (Table 4). The experiment determines three cysteines in the protein and demonstrates that no cystines are present, in agreement with three cysteines in the sequence of the protein. These cysteines can be blocked by either alkylation or oxidation (Table 4).

### 2.5. Determination of the ZnT8c Ni(II) Complex Stoichiometry with Fluorescent (Fluozin-3) and Colorimetric (Zincon) Indicators

The affinities of the proteins’ binding sites for Ni(II) ions relative to chelating agents were estimated by competition of ZnT8c with FluoZin-3 or Zincon. Both agents are routinely used to measure Zn(II) ions but they also can be used to measure Ni(II) ions. The reported affinities of FluoZin-3 for nickel and zinc are: FluoZin-3/Ni^2+^ 2.85 ± 0.03 nM [28], FluoZin-3/Zn^2+^ 9.1 ± 0.4 nM [29]. Zn^2+^ binds Zincon with a dissociation constant of 7 ± 1 µM, which is weaker than that for Ni^2+^ ions [30,31].

**Figure 3:**
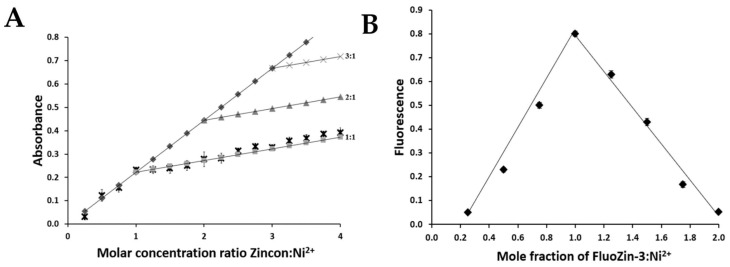
Assessment of the Zincon-Ni(II) and FluoZin-3-Ni(II) complex stoichiometry at pH 7.0. (**A**) Complementary Approach. The molar concentration of Ni(II) ions was kept constant at 25 μM and the total molar concentration of Zincon was continuously varied from 6 to 100 μM. The line with the grey diamonds corresponds to the absorbance of the complex formed after an excess of nickel was added. The other lines represent the theoretical absorbances expected if the stoichiometries were 1:1 (squares), 2:1 (triangles) or 3:1 (crosses). The comparison between the measured absorbance (black crosses) with the theoretical lines establishes a stoichiometry of 1:1. (**B**) Job’s method. The ratios refer to chelating agent/nickel. The total molar concentration of FluoZin-3 and Ni(II) was 100 μM. The lines converge on a molar ratio of 1:1. The assessment was performed with three independent stock solutions.

To investigate the stoichiometry of nickel binding to ZnT8c in competition with FluoZin-3 or Zincon, two independent methods were used: a classical approach (Job’s method) and a relatively new one (the complementary approach). Zincon or FluoZin-3 was mixed with nickel ions in the buffer. Addition of Ni(II) ions to Zincon at pH 6.0, 7.0, and 8.0 resulted in shifts of the absorbance maxima from 488 nm (Zincon alone) to 653 nm (Zincon-nickel complex). Fluorescence of FluoZin-3 (λex = 480 nm and λem = 520 nm) showed a small shift in the position of the excitation wavelength (λex = 490 nm) at all pH values examined, demonstrating the Ni(II) complex formation. Under all tested conditions a 1:1 ± 0.1 stoichiometry was detected in the Zincon-Ni^2+^ or the FluoZin-3-Ni^2+^ system (Figure 3).

**Figure 4:**
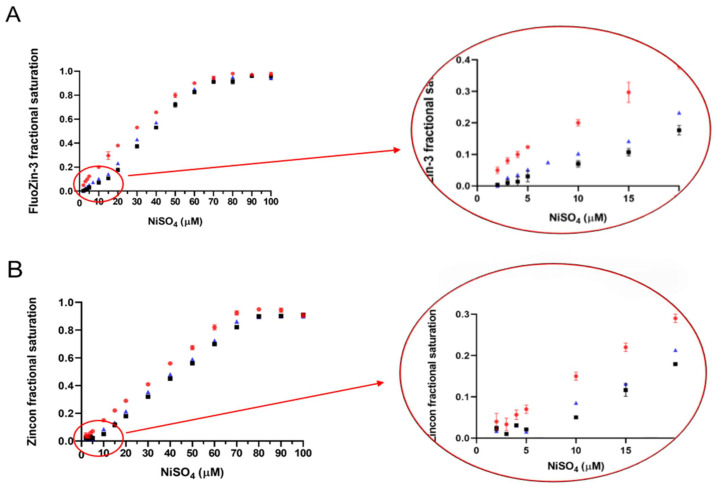

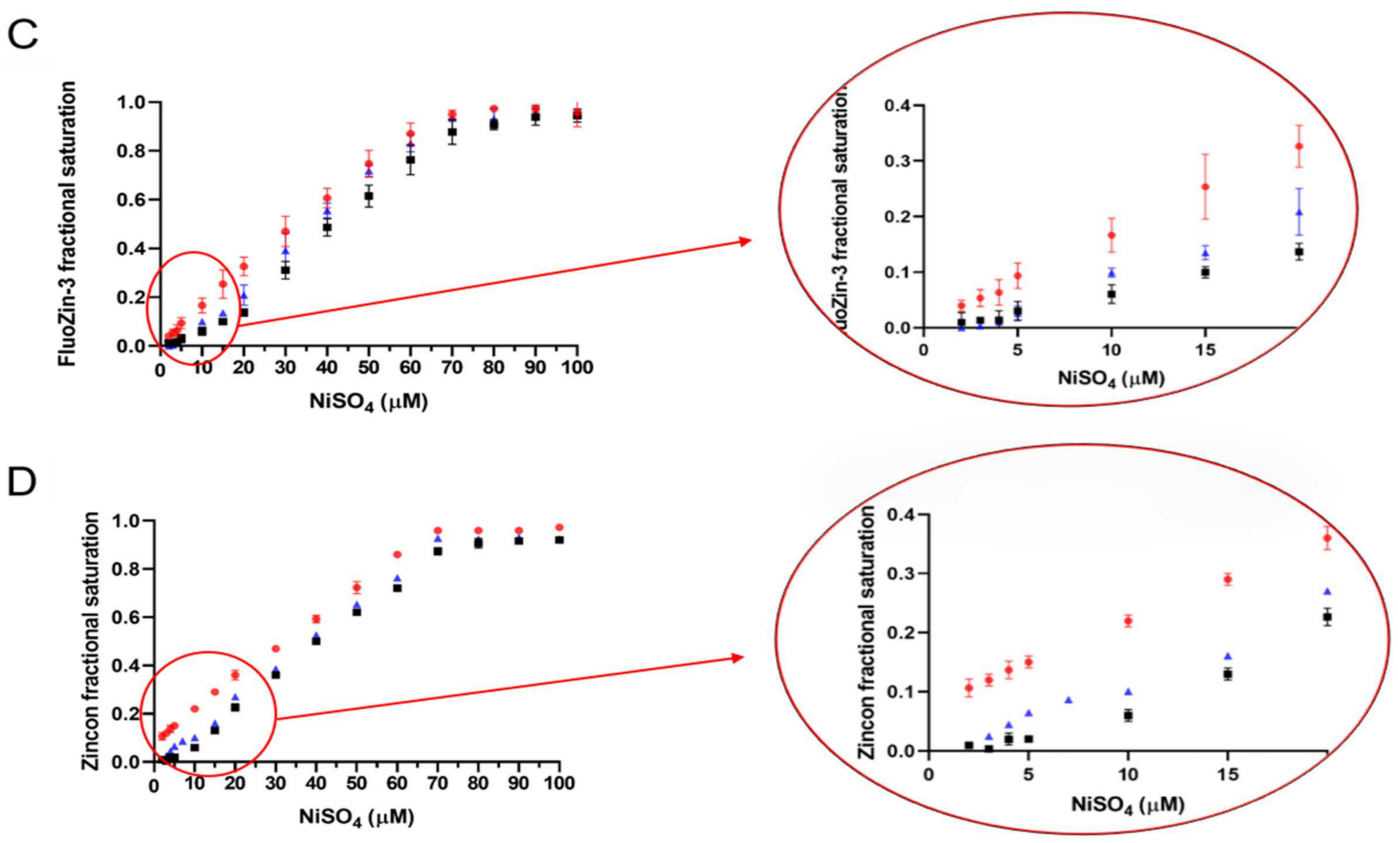
Nickel affinity of the two ZnT8 C-terminal domain (CTD) variants before and after alkylation. ZnT8cW. Nickel binding in competition with FluoZin-3 (**A**) and Zincon (**B**). Measuring fluorescence at 515 nm and absorbance at 653 nm, 70 μM NiSO_4_ saturates 70 μM FluoZin-3/Zincon in 50 mM HEPES (4-(2-hydroxymethyl)-1-piperazineethanesulfonic acid), 300 mM NaCl, 100 mM sucrose, pH 8 in agreement with the stoichiometry of the complex (red circles). In competition with 5 µM ZnT8cW, no signals at 515 nm or 653 nm are detected until 10 μM NiSO_4_ is added, revealing two high-affinity nickel binding sites in ZnT8cW which outcompete FluoZin-3/Zincon (black squares). When ZnT8cW is incubated with iodoacetamide for 1 h prior to the FluoZin-3/Zincon competition assay, only 5 μM NiSO_4_ is required to elicit the initial signals at 515 and 653 nm and the stoichiometry decreases to 1 (blue triangles). ZnT8cR. Nickel binding in competition with FluoZin-3 (**C**) and Zincon (**D**). NiSO_4_ titration of FluoZin-3/Zincon alone in HEPES buffer (red circles), in competition with ZnT8cR (black squares), and in competition with ZnT8cR modified with iodoacetamide (blue triangles), demonstrates that ZnT8cR also contains two high affinity nickel binding sites and that one binding site is blocked by alkylation.

ICP-MS analyses showed that both ZnT8c proteins contained 0.50 ± 0.05 (ZnT8cW) and 0.60 ± 0.1 (ZnT8cR) nickel per monomer following purification (Table 3 and Table 5). Each ZnT8c variant (5 μM) then was incubated with Ni^2+^ (from 0 to 100 µM) with or without 15 mM iodoacetamide and underwent gel filtration to remove excess Ni^2+^. Stoichiometry calculations are based on the increasing fluorescence/absorbance when 10 μM Ni^2+^ was added in competition with 5 μM of protein (Figure 4). ICP-MS analysis also was performed after adding 10 µM Ni^2+^ in the presence and absence of iodoacetamide and the stoichiometries calculated (Table 5).

The competition assays and ICP-MS analyses demonstrate that both ZnT8c proteins bind two Ni^2+^ ions per monomer in high affinity sites (Figure 4 and Table 5). Once the cysteines are blocked, the nickel:protein stoichiometry decreases from 2:1 to 1:1. The data from both direct metal analyses and competition assays therefore show that the cysteines at the C-terminus are one of the two binding sites for nickel.

### 2.6. Circular Dichroism (CD) Spectroscopy

**Figure 5:**
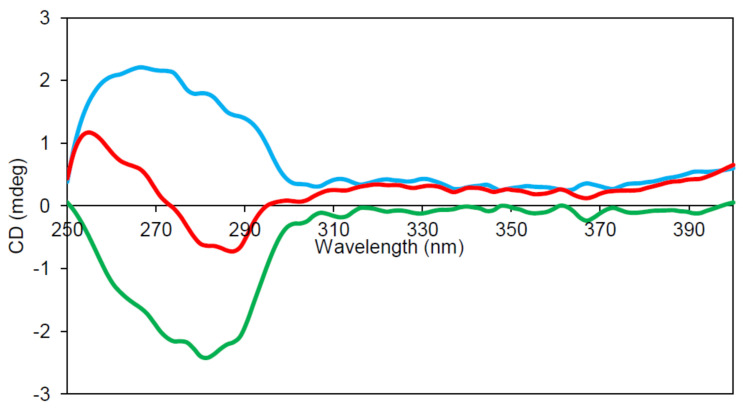
Near-UV circular dichroism (CD) of the two ZnT8c variants. Representative (n = 3) near-UV CD spectra of 0.4 mg/mL ZnT8cR (blue) and ZnT8cW (green) in 50 mM Tris, 300 mM NaCl, 100 mM sucrose, 100 µM Tris(2-carboxyethyl)phosphine hydrochloride (TCEP), pH 8. Subtracting the ZnT8cR spectrum from that of ZnT8cW provides the expected CD of W325 in the protein alone (red).

**Figure 6:**
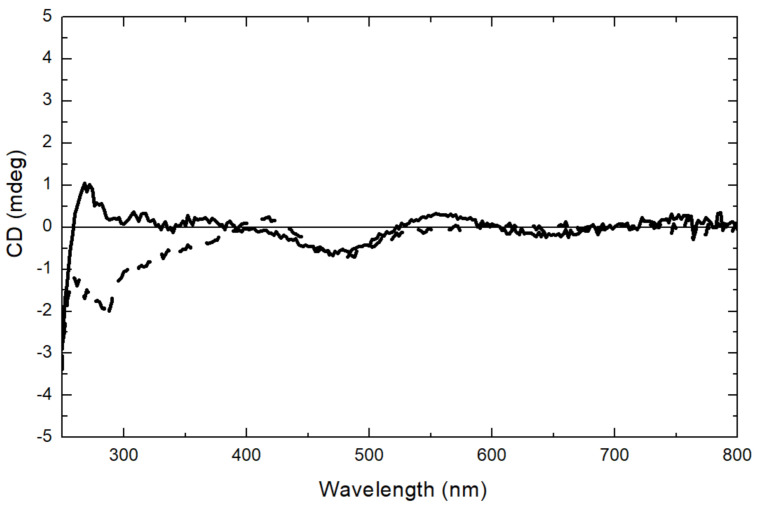
CD spectra of ZnT8c with Ni^2+^ excess. ZnT8cR + 0.1 mM NiSO_4_ (black line) and ZnT8cW + 0.1 mM of NiSO_4_ (dotted line) in 50 mM Tris, 300 mM NaCl, 100 mM sucrose, 100 µM TCEP, pH 8, 10 mm pathlength. The concentrations of the ZnT8c proteins were 35 µM. Corrected for buffer baseline.

The spectra in the region 260–320 nm arise from the aromatic amino acids. Each of the amino acids has a characteristic wavelength profile. ZnT8cR contains four Tyr, two Phe, and one Trp residue; ZnT8cW contains an extra Trp. Subtraction of the apo-ZnT8cR near-UV CD spectrum from that of apo-ZnT8cW reveals a single negative peak at approximately 285 nm (Figure 5), within the expected range for Trp residues. The differences in the near UV CD of the two proteins demonstrate that the environment of the extra Trp in ZnT8cW can be investigated by difference spectroscopy. Since we observed light absorption of the isolated proteins above 300 nm, we suspected that the absorbance is indicative of bound Ni(II) ions, which can be used as a spectroscopic probe of the zinc-binding sites in the CTD. To gain more insight into the coordination, we extended the CD spectra to the visible region of the spectrum for both species. A positive CD band at 312 nm and a negative one at 484 nm were observed. The latter is also indicative of the presence of nickel and becomes more pronounced when the protein’s binding sites are saturated by adding 0.1 mM Ni^2+^. A small difference in the CD spectra of the two variants was observed (Figure 6) and the negative CD band at 484 nm of ZnT8cW + 0.1 mM of Ni^2+^ was observed as well. Moreover, only the CD spectrum of ZnT8cR + 0.1 mM of Ni^2+^ shows a positive band around 550 nm and a negative band at around 650 nm. To determine if the ZnT8c Ni^2+^ binding site has free coordinating ligands, NaN_3_ was added to a final concentration of 0.3 mM. This experiment is based on the expectation that, if there were a water or non-protein ligand bound to ZnT8c, the displacement of such a ligand by binding to a nitrogen donor from the azide would provoke a change in the CD spectrum, as documented for azide binding to Ni^2+^ in the active site of horse liver alcohol dehydrogenase [32]. The spectra did not change substantially, indicating that the Ni(II) site(s) were coordinatively saturated by ligand donors from amino acids and not accessible. These experiments reveal differences in the Ni(II) binding sites in the two variants and establish that Ni(II) ions can be used to examine structural differences between the two proteins.

### 2.7. Potentiometric Characterization of Zinc Binding to N-Terminal and C-Terminal Peptides of ZnT8

As the N-terminus, which is part of the transmembrane domain, has a HCH metal-binding motif, we synthesised an N-terminal fragment of ZnT8 (Ac-MYHCHSGS-NH_2_) and tested its ability to bind zinc ions. Though the peptide contains a tyrosine and is thus amenable to fluorescence spectroscopy, the most reliable data were obtained using the potentiometry method. The protonation and stability constants for the peptide and the complex with Zn(II) are given in Table 6.

**Figure 7:**
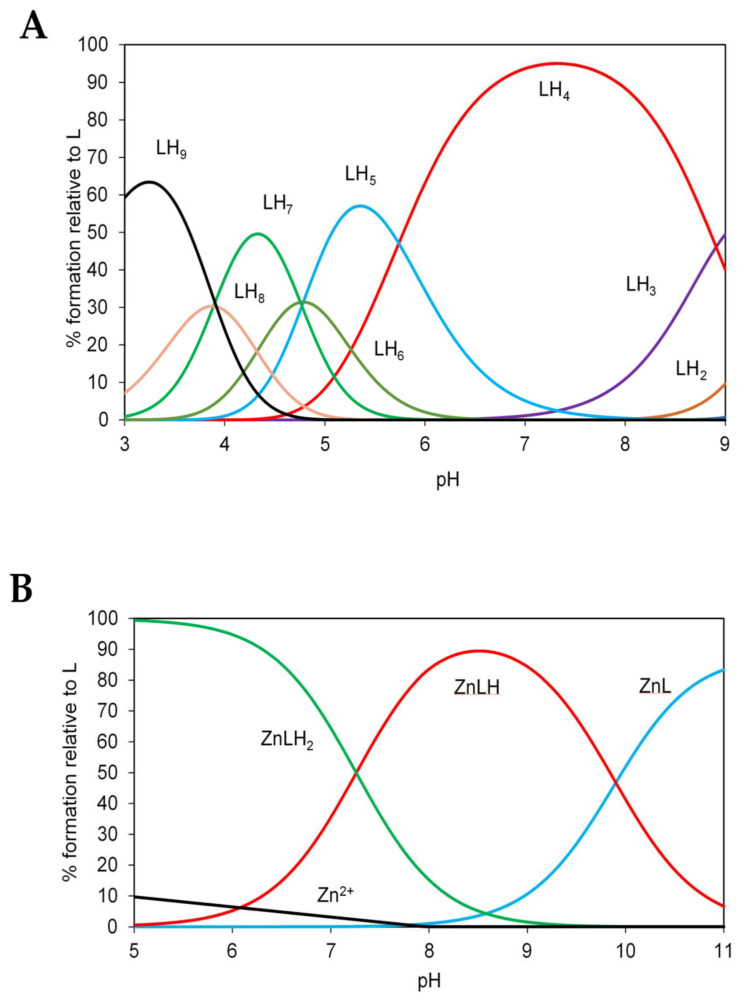
Speciation diagrams for the ZnT8 11-residue C-terminal peptide. (**A**) Protonation of 180 µM peptide (denoted L) during titration with NaOH according to data in Table 6. At pH 7.4 the dominant species is LH_4_ (red). (**B**) Modeling of 180 µM peptide with 200 µM Zn^2+^ using the stability constants in Table 8 reveals that at pH 7.4 there is a mix of 58% ZnLH (red) and 42% ZnLH_2_ (green) relative to the peptide concentration. In both experiments the ionic strength is 0.1 M and the temperature is 25 °C.

Remarkably, the peptide p*K*_a_ value for one of the histidines is lower than expected and the p*K*_a_ of the tyrosine is higher than 10. These findings suggest additional interactions between these two residues. Zn(II) binds to the ZnT8 N-terminal fragment through the Cys and at least one of the His residues. The conditional dissociation constant value of *K*_d_ = 0.56 ± 0.03 μM (pH 7.4) is too weak to indicate a significant biological role of the N-terminal peptide alone. The calculation was made using the competitivity index (CI) approach based on the potentiometric stability constants [33]. In view of our previous investigations showing the participation of the C-terminal cysteines in zinc coordination [21] and the observation here that the cysteines also interact with Ni(II) ions, we also synthesized an 11-residue C-terminal peptide of human ZnT8, sequence Ac-PDCLFCEDPCD (denoted ‘L’ for ligand in Figure 7 and Table 7 and Table 8), verified its mass using mass spectrometry, and characterized its protonation and zinc binding. Visible spectroscopy measurements indicated that concentrations of peptide greater than 0.2 mM were insoluble in the HNO_3_/KNO_3_ reaction solution. Therefore, 180 μM peptide was prepared in 7.5 mL of 0.4 mM HNO_3_/99.6 mM KNO_3_ solution. The protonation constants from potentiometric titrations of the peptide with 0.1 M NaOH (carbon dioxide free) indicated that the major protonated species of the C-terminal apo-peptide is LH_4_ at pH 7.4 (Table 7; Figure 7A). Comparing the modelled log *K* protonation constants to a physiological pH of 7.4, the first four protonations (LH–LH_4_) occur at a pH higher than 7.4. According to assignments provided in Table 7, the three cysteine residue thiols (p*K*_a_ = 8.9–10.1) and the N-terminal amine are protonated at pH 7.4. To determine the zinc affinity of the peptide, separate peptide samples were incubated with 0.5, 0.65, 0.8 and 0.9 molar equivalents of Zn^2+^, and titrated with 0.1 M NaOH as for the apo-peptide. Modeling of these data yielded stability constants of the peptide-Zn^2+^ complex (Table 8). The stability constant of the first peptide ligand (ZnL, log *K* 15.98) is much higher than that of the second ligand (ZnL_2_, log *K* 7.35). Therefore, the ZnL_2_ complex does not form to a significant degree and can be ignored during modeling of the physiological speciation. The zinc-ligand species distribution diagram indicates a mix of 58% ZnLH and 42% ZnLH_2_ at pH 7.4 (Figure 7B). The effective log *K* of the peptide for Zn(II) ions can be calculated using the CI approach [33]. The CI calculated for the Zn/L system is 8.24, hence the *K*_d_ of the C-terminal 11-residue peptide of ZnT8 for Zn^2+^ at pH 7.4 is 10^−8.24^ M (5.8 nM).

## 3. Discussion

**Figure 8:**
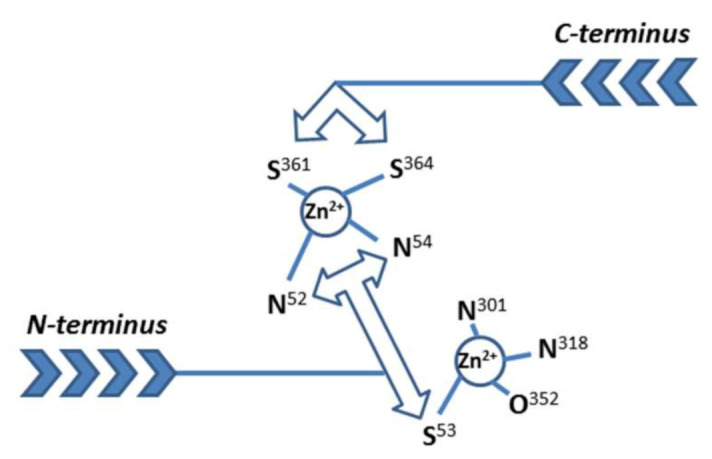
The two Zn^2+^-binding sites in the cytosolic C-terminal domain (CTD) of human ZnT8. The presentation is based on a recent Cryo-EM structure of the entire protein [20]. One metal ion is bound by two N-donors from histidines (301 & 318) and one O-donor from glutamate (352). An S-donor from cysteine (53) from the N-terminus (not present in the CTD) complements a tetrahedral binding geometry. The second site is assembled from donors of the C- and N-termini only: Two S-donors from cysteines (361 & 364) from the C-terminus and two N-donors from histidines (52 & 54) from the N-terminus. The N-terminal tripartite motif with adjacent metal-coordinating amino acid side chains (HCH) “seals off” the metal sites [20]. Both termini have to swing into the site to effect coordination of the second metal ion.

The vesicular subfamily of mammalian ZnTs (ZnT2-4, 8) mediates the transport of zinc from the cytosol into functionally critical intracellular secretory granules. While the general architecture of the transporters themselves have been predicted from bacterial homologues for some time, and the Zn^2+^/H^+^ antiport transport mechanism described in previous transport assays, answers to several fundamental biochemical issues about these transporters remain elusive. The overarching problem is how these proteins access the tightly controlled cytosolic pool of zinc, most of which is bound to proteins with high affinity. In the bacterial proteins, the CTD acts as an allosteric gatekeeper, activating the transporter upon detection of a cytoplasmic Zn(II) ion threshold. Our analysis showed that essential residues are missing from the CTD in the mammalian proteins [21]. Therefore, the aim of our work was to answer the general questions of why the mammalian ZnT cytosolic domain binds metal ions, whether it senses zinc ions for export, and if so, at which concentrations, and how selective the binding sites for metal ions are. Specifically, for ZnT8, we are also investigating how the W/R mutation at the apex of the CTD of ZnT8 affects metal transport far away in the transmembrane domain and thus can modulate the risk to developing type 2 diabetes. Two recent cryo-EM structures of full-length human ZnT8 describe its overall structure [19,20]. One of the two investigations provides a model at relatively high resolution (3–5 Å) and important new information on metal binding [20]. The CTD indeed adopts the ferredoxin-type αβ-fold predicted from CD studies [21]. As we also predicted, the cryo-EM structure revealed that full-length ZnT8 binds two zinc ions in the CTD, the same number as in *E. coli* YiiP. However, the site location and coordinating residues in the ZnT8 CTD binding sites are partially distinct from those in YiiP. One difference is the participation of two cysteines from the C-terminus of the CTD. Remarkably, the N-terminal domain (NTD) that is present only in the full-length ZnT8 protein also provides ligands to complement the coordination spheres of both zinc binding sites in the CTD [20]. The NTD contains an HCH motif, in which all three ligands are used in a three-pronged mode of binding. The first binding site in the CTD is formed of two histidines (H301 & 318) and one glutamate (E352) provided by the CTD, and one cysteine (C53) provided by the NTD [20]. This site is analogous to one of the two sites in the CTD of *E. coli* YiiP, with H301, H318 and E352 being analogous to H232, H248 and D285 in YiiP, as we predicted previously [21]. However, the fourth ligand providing the tetrahedral geometry in YiiP is a water molecule, as opposed to the NTD C53 in ZnT8c (Figure 8) [20]. Therefore, the solvent accessibility of this bound zinc ion is different between the bacterial and human proteins. The ligands providing the second zinc binding site in the CTD of *E. coli* YiiP are not conserved in mammalian ZnTs [21]. Thus, the second metal binding site in ZnT8c is novel and is formed of two histidines (H52 & 54) from the NTD which complement the two cysteine ligands (C361 & 364) from the CTD (Figure 8) [20]. Thus, remarkably, this site is formed entirely from ligands stemming from both termini of the protein. The recent ZnT8 cryo-EM structure at the high resolution therefore verifies our previous and current results showing that the C-terminal cysteine residues in ZnT8c provide a metal binding site [21] and indicate that it is important for the function of the full-length protein. The functional implications of these ZnT8c metal binding sites are vast. The C-terminal cysteines are unique for the vesicular ZnTs, and their absence in the CDF/ZnT plasma membrane transporters in both prokarya and eukarya led to the prediction that non-vesicular ZnTs contain only one metal binding site in their CTD [21]. The vesicular transporters must acquire zinc from an unknown donor in a tightly zinc-buffered cytosol, whereas the exporters remove an excess/surplus of zinc; the novel CTD metal ligands from the C- and N-termini in the vesicular subfamily, as exemplified here by ZnT8, suggest that at least one zinc ion is acquired through the C-terminal cysteines. Instead of a ligand from the other subunit bridging the binuclear metal site, as in the dimer of YiiP, the tripartite N-terminal HCH motif in ZnT8 serves as a brace for linking the two metal sites. Thus far, such a coordination of metal sites is unique in bioinorganic chemistry and it is the first example of zinc/sulfur coordination chemistry in eukaryotic zinc transporter biology.

The involvement of cysteine residues in both CTD metal binding sites indicates that zinc binding is dependent on the cellular redox balance. This observation is significant as oxidative stress in pancreatic β-cells, which is recognised as a fundamental aspect of type 2 diabetes pathology [34], is expected to affect zinc binding to the CTD. Since the CTD binds two metal ions with relatively high affinity in the absence of the TMD with its N-terminus, it is now important to determine the contribution of the N-terminal HCH motif to affinity and function. It is noteworthy that investigations with a synthetic peptide of human ZnT3 bearing the HCH motif already have shown relatively high affinity zinc binding, a significant preference of zinc over nickel, and has implicated the motif in the function of the vesicular transporters [35]. The rather disordered C-terminal tail of ZnT8 (and by inference that of other vesicular ZnTs) may serve as a swinging arm that acquires zinc through coordination to its three cysteines and then swings over to form the metal site with the other ligands. The single digit nanomolar affinity of the C-terminal peptide for zinc supports such a role. The flexibility of the C-terminal tail and the low sigma value of zinc in the site with the two cysteines from the tail also support such coordination dynamics [20]. “Putting a brace on” with the N-terminal HCH motif would then essentially capture zinc ions in this site. The important feature of such coordination dynamics is that the N-terminus connects to TM1, which together with TM2,4 &5 is critical for the dynamics of the protein in alternating between the inward and outward conformation for transport [20].

Owing to its filled d-shell, the Zn(II) ion is spectroscopically silent for many types of spectroscopies. One approach to overcome this experimental challenge is to employ transition metal ions with specific spectroscopic signatures to examine zinc-binding sites in proteins and to gain information about the coordination environments [36]. After noting that Ni(II) ions bind to the cytosolic domain of the zinc transporter ZnT8 (ZnT8c) during purification, we employed nickel ions to probe the metal-binding properties of the two variants and to gain further insights in the coordination environments of zinc. The ZnT8c variants with Ni(II) ions bound have different CD spectra, supporting our previous conclusion that the differences in metal binding impart slightly different properties on the protein. The spectra are very similar to the complex of Ni^2+^-substituted alcohol dehydrogenase, which has an S_2_N_2_ coordination environment in its complex with an external N-donor ligand [32]. Similarity is also evident based on a comparison with Ni(II)-glutathione complexes [37]. The Ni(II) sites in both ZnT8c variants are low-spin square-planar or square-pyramidal as evidenced by a clear band at 490 nm, and represent thiol coordination as evidenced by a ligand-to-metal charge transfer transition at 290 nm. The site geometries are significantly different in the two variants, because of the opposite signs of the 290 nm band and the presence of additional bands in the individual cases. The three d-d electronic transitions in ZnT8cR suggest a more significant deviation from the planarity of the complex, while the low-energy CT band present only in ZnT8cW—apparently an S(π) → Ni(II) transition—suggests different arrangements of sulfur donors in space. A second approach to characterize metal binding is to use chromophoric or fluorogenic chelating agents. We previously used the chromophoric chelating agent Zincon in competition with ZnT8c to investigate the affinity and stoichiometry of zinc binding [21]. Here, we employ this reagent and a fluorogenic Zn(II) chelating agent, FluoZin-3, to measure the binding of Ni(II) ions and probe the participation of sulfur ligands from cysteines following their chemical modification, extending the use of these reagents in (inorganic) chemical biology. Like Zn(II) binding, there is one Ni(II) binding site that is abolished when the cysteines are modified, leaving one site, which could be either the N-terminal 6xHis tag, which binds Ni(II) ions with an affinity of 700 nM [38], or the other CTD site, or could reflect a distribution of nickel between both. The CTD forms a tetramer when the N-terminal 6xHis tag is removed [39]. In agreement with the investigations of others, we find that the CTD with the tag forms a dimer [19,21]. Investigating Ni(II) binding is also important for the biology of this transporter, because there are Ni^2+^ transporters in the CDF family [40] and nickel has been found in human islets [41], and therefore selectivities of the metal binding sites need to be understood. It is not only an issue of whether ZnT8 transports nickel, but also how the binding of other metal ions in the CTD affects zinc transport. Mismetalation in the CTD could modulate transporter function and have significant consequences on islet biology and glucose homeostasis. A case in point is cadmium. A single substitution of an Asp in the zinc transport site of YiiP to a His in human ZnT confers selectivity for zinc over cadmium [42]. However, a corollary of sulfur coordination in the metals sites is that cadmium, a thiophilic cation, is likely to bind in the CTD and affect the function of ZnT8, as it has been shown that cadmium accumulates in mouse β-cells and impairs their function [43]. In addition, the two variants have slightly different properties in their interactions with metal ions and it will be interesting to find out how this difference relates to the diabetes T2 risk and the antigenicity of the protein in diabetes T1.

## 4. Material and Methods

### 4.1. Materials

HEPES, iodoacetamide, Zincon sodium salt, NaCl, hydrogen peroxide, ZnCl_2_, KCl, NiSO_4_, gallium (99.9995%), NaN_3_, HNO_3_ 70%, methanol, acetic acid, and ethanol were purchased from Sigma Aldrich (Merck Life Science UK Ltd., Gillingham, UK); Tris hydrochloride (TRIS/HCl) from MP Biomedicals (Fisher Scientific UK, Loughborough Leicester, UK); 5,5′-dithio-bis-(2-nitrobenzoic acid) (DTNB; Ellman’s reagent) from Invitrogen (Thermo Fisher Scientific UK, Ashford, UK); ≥18.2 MΩ cm ultrapure water was obtained from an ELGA LabWater UK system (High Wycombe, UK).

### 4.2. Protein Purification

The ZnT8 CTDs (ZnT8c protein) were expressed and purified according to our established protocol [21]. Denaturing sodium dodecyl sulfate-polyacrylamide gel electrophoresis (SDS-PAGE) was performed according to the Invitrogen NuPAGE® specifications. Briefly, 5 μL of protein sample (5–20 μg protein) were mixed with 10 μL of sample loading buffer (Invitrogen) and heated at 80 °C for 14 min. Samples were loaded into precast NuPAGE Novex 14% Bis-Tris 1.0 mm minigels (Invitrogen) with 5 μL of Pre-stained SDS-PAGE Standards (Bio-Rad, Watford Hertfordshire, UK). Electrophoresis was performed at 25 °C for approximately 55 min using a constant voltage (200 V) in a solution of NuPAGE SDS running buffer (Invitrogen) until the dye front reached the end of the 60 mm gel. Gels were washed three times in ultrapure water for 5 min, stained twice in 100 mL of fixing solution (40% methanol and 60% acetic acid), incubated in 100 mL of Coomassie R250 solution for 1 h and placed on a shaker for 30 min. Dilute size exclusion fractions were concentrated using 15 mL 3 kDa molecular mass cut-off centrifugal concentrators (Merck Millipore, Merck Life Science UK Ltd., Gillingham, UK). The concentration of pure ZnT8c protein was checked both spectroscopically using a Nanodrop 2000 (Thermo Scientific, Thermo Fisher Scientific UK, Ashford Kent, UK) instrument and spectrophotometrically using a modified Bradford protein assay. The extinction coefficients of 13,980 and 8480 M^−1^ cm^−1^ for ZnT8cW and ZnT8cR, respectively, were calculated from the primary protein sequences [21].

### 4.3. Elemental Analysis by Inductively Coupled Plasma-Mass Spectrometry

Samples were analyzed using ICP-MS (PerkinElmer NexION 350D, Seer Green, Bucks, UK) equipped with a glass Meinhard nebulizer. The ICP-MS settings were: gas flow 1 L/min; auxiliary gas flow 1.2 L/min; plasma flow 18 L/min; RF power 1600 W; cell gas flow He 4.1 mL/min. Elemental standards (Zn and Ni) in 2% (*v*/*v*) HNO_3_ were used for calibration and detected as ^66^Zn and ^60^Ni isotopes. Prior to sample analysis, calibration curves for each element were constructed over the range 0.1–1000 µg/L. Linear regression correlation coefficients (R^2^) better than 0.9990 were obtained in all cases. A digestion procedure commonly applied to biological specimen was used: samples (100 µL) were digested with 400 µL of 75% HNO_3_ and heated at 60 °C overnight, and then diluted for analysis with ultrapure water to a final HNO_3_ concentration of 2%.

### 4.4. Elemental Analysis by Total Reflection X-ray Fluorescence 

TXRF measurements were performed with a Bruker S2 PICOFOX TXRF (Bruker AXS Microanalysis GmbH, Berlin, Germany) benchtop instrument equipped with molybdenum tube and silicon drift detector. The excitation source operated at 40 kV and 650 µA. A gallium solution (10 mg/L) in nitric acid was used as internal standard for quantification. For each sample, 40 µL of solution was prepared for TXRF analysis. Specimens were prepared by depositing 10 μL of sample solution on the quartz glass sample carrier and drying at 50 °C on a heating plate. Each specimen was measured for 10 min; duplicates of each sample were performed. TXRF spectra were inspected, all elements identified, and deconvoluted using the PICOFOX^TM^ software. Elemental concentrations of the different elements were calculated by reference to the Ga standard in each sample.

### 4.5. Determination of Metal Binding with Competing Chelating Agents

The affinities of the ZnT8c sites for Ni^2+^ were estimated by using two indicators: FluoZin-3 and Zincon. The stoichiometry between Ni^2+^ and either FluoZin-3 or Zincon was investigated by using two methods: Job’s method [44] and the Complementary Approach [45]. The stoichiometries of the metal/protein complexes were obtained from a competition assay used to determine the zinc content of the protein [21]. To examine whether the metal ion is forming a complex with the indicator, a methanolic solution of FluoZin-3 or Zincon was mixed with an aqueous solution of metal ions for 1 min at different molar concentration ratios ranging from 1:4 to 1:50 (indicator:metal) at different pH values. The concentration of FluoZin-3 or Zincon ranged from 20 to 50 µM while that of nickel was kept constant at 300 µM. The blank for indicator and nickel complex measurements was composed of methanol and water in a 1:1 ratio.

### 4.6. Job’s Method

Job’s method, also known as the method of continuous variation, is a simple analytical approach which determines the stoichiometry of two interacting components. In this method, the total molar concentration of two reactants is kept constant while their molar concentration ratios are continuously varied throughout the series of samples. According to Job’s method, a peak in spectroscopic readout at a specific concentration ratio corresponds to the stoichiometry of the complex formed. In this experiment, an aqueous solution of nickel was mixed with a methanolic solution of the substance to be tested (FluoZin-3/Zincon) at different molar concentration ratios ranging from 1:3 to 6:1 (indicator:metal) and after 3 min absorption spectra were measured against a blank composed of methanol and water in a 1:2 ratio.

### 4.7. Complementary Approach

For the complementary approach the molar concentration of indicator was continuously changed, while the concentration of nickel was kept constant in the samples with different molar concentration ratios ranging from 1:3 to 6:1 (indicator:metal). The concentration of the metal ion was 25 µM, while that of FluoZin-3 or Zincon were varied from 6 to 100 µM. The blank composition was the same as in the case of Job’s method. Calculation of the complex stoichiometry is based on our mathematical approach [45]. Briefly, molar absorption coefficients for FluoZin-3 or Zincon and their complexes with Ni(II) were calculated by measuring a series of different concentrations of FluoZin-3 or Zincon with or without the metal ion. Based on these coefficients, theoretical lines were constructed representing the absorbances of the most probable integer stoichiometries and matched with measured data of the different indicator:nickel ratios to determine the stoichiometry.

### 4.8. FluoZin-3 as a Fluorophore to Detect Nickel(II)

The principle of this method is based on an increase in fluorescence when the indicator, which is generally employed for the determination of Zn(II), also binds Ni(II). The complex formation was determined spectrofluorometrically (Fluoroskan Ascent CF, Labsystems, ThermoFisher) using 96-well plates (Thermo Scientific). All experiments were performed with a buffer containing 50 mM HEPES, 500 mM KCl, and 100 mM sucrose at pH values ranging from 6.0 to 8.0. A stock solution of 100 mM NiSO_4_ was prepared in 10 mL of water and used for the experiments upon dilution with buffer. A stock solution of 1 mM indicator in methanol was diluted with buffer to a final concentration of 70 µM. The indicator and the metal ions were mixed in buffer for 2 min, the samples incubated for 1 min at 25 °C in the dark and electronic absorption spectra measured (300–800 nm). Fluorescence was measured at λem = 520 nm and λex = 480 nm.

### 4.9. Zincon as a Chromophore to Detect Nickel(II)

Zincon, a complexing agent generally employed for the colorimetric determination of Zn(II) [46], can also be employed for the determination of Ni(II). The concentration of free Zincon was measured at 488 nm with a Jenway 7315 spectrophotometer (Chelmsford Essex, UK) using the extinction coefficient ε_488_ = 26,900 M^−1^ cm^−1^ and a 0.5 cm path length quartz cuvette [21]. All experiments were performed in 50 mM HEPES, 300 mM NaCl, and 100 mM sucrose at pH values ranging from 6.0 to 8.0 with a methanolic Zincon solution at a final concentration of 70 µM. Concentrations of Ni(II) added were from 10 to 100 µM. Absorbance of the Zincon-Ni^2+^ complex was determined at 653 nm.

### 4.10. Thiol Assays

A 5,5′-dithio-bis(2-nitrobenzoic acid) (DTNB) assay was used to quantify the free sulfhydryls at the C-terminus of ZnT8c proteins. The protein samples were adjusted to a final volume of 2 mL and to a final protein concentration of 2 µM by using 500 mM HEPES pH 8, 300 mM NaCl and 100 mM sucrose. After the addition of DTNB to a final concentration of 1 mM the samples were vortexed for 1 min and incubated at 20 °C for 15 min. The production of 2-nitro-5-thiobenzoate (TNB) was measured at 412 nm, using the extinction coefficient ε_412_ = 14,150 M^−1^ cm^−1^. The blank contained deionized water. Iodoacetamide and hydrogen peroxide were used to alkylate or oxidize the sulfhydryls on the cysteine side chains. Alkylation was confirmed by measuring no free sulfhydryl in the protein with DTNB. For both reactants, 0.5 M stock solutions were prepared in ultrapure water. Protein samples were incubated with 15 mM of either iodoacetamide or hydrogen peroxide for 1 h at 20 °C.

### 4.11. Protein Competition Assays

Competition assays were performed by using both indicators (FluoZin-3 and Zincon). ZnT8c protein (5 μM) was added to the mixture with the indicators, both at working concentrations of 70 μM. The solutions were mixed for 2 min and the absorbance/fluorescence measured. Binding stoichiometries were analyzed by extrapolation of the linear portion of the titration curves and comparison with the buffer-only controls. The three cysteines at the C-terminus of ZnT8c were acetylated using iodoacetamide to evaluate whether they contribute to metal binding. Iodoacetamide (stock solution 0.5 M in water) was added to the mixture of 5 μM ZnT8c and incubated for 1 h at 20 °C, prior to the competition assays. Statistical analyses were performed using the software GraphPad Prism version 8 for Windows (GraphPad Software, La Jolla, CA, USA).

### 4.12. Far-UV Absorbance and Circular Dichroism Spectroscopy

Protein UV and CD spectra were acquired on an Chirascan Plus spectrometer (Applied Photophysics, Leatherhead, UK). Protein samples of either ZnT8c variant were diluted to 35 μM in 10 mM HEPES, pH 8, 60 mM NaCl, 20 mM sucrose and incubated with 0–2 molar equivalents of Zn^2+^ for 20 min at 21 °C. A 10 mm Quartz Suprasil rectangular cuvette (Starna Scientific, Ltd., Ilford, UK) was employed in the spectral region 230–800 nm. The instrument was flushed continuously with nitrogen gas. The following parameters were employed: 2 nm spectral bandwidth, 1 or 2 nm step size and 1 or 1.5 s accumulation time-per-point. The Near-UV-CD spectra were smoothed using Chirascan Pro-Data Viewer software v4.2.15 (Applied Photophysics) with a Savitsky-Golay smoothing factor of 4. The UV and CD spectra were corrected with appropriate buffer solutions. Unless otherwise stated, all spectra were measured at 23 °C. Data are presented using Origin V6 (OriginLab Corp.). The cuvette was washed with a mixture of concentrated nitric acid and ethanol (50:50, *v*/*v*) and dried using nitrogen gas before and between sample measurements to prevent cross contamination. Measurement of each sample was repeated after 10 min to determine whether spectra changed due to protein aggregation.

### 4.13. C-Terminal and N-Terminal Peptide Syntheses

For peptide synthesis, the dedicated Fmoc protected amino acids and 2-(1H-benzotriazole-1-yl)-1,1,3,3-tetramethyluronium hexafluorophosphate (HBTU) were purchased from NovaBiochem (Merck Sp., Warszawa, Poland). The TentaGel S RAM and TentaGel S PHB-Lys(Boc)-Fmoc resins were obtained from RAPP Polymere (Tübingen, Germany). The solvents of piperidine, triisopropylsilane (TIS), *N*,*N*-diisopropylethylamine (DIPEA), 1,2-ethanedithiol (EDT) and trifluoroacetic acid (TFA) were received from Sigma (Sigma-Aldrich Sp., Poznan, Poland). Dimethylformamide (DMF) was purchased from Carl Roth (Karlsruhe, Germany), acetonitrile from Avantor (VWR Sp., Gdańsk, Poland) and diethyl ether from Chempur Feinchemikalien (Karlsruhe, Germany). The Ac-MYHCHSGS-NH2 (ZnT8N) and Ac-PDCLFCEDPCD (ZnT8C) peptides were synthesized on a Liberty 1 automated peptide synthesizer (CEM Corporation, Buckingham, UK) according to the standard solid-phase Fmoc strategy [47]. The acetylation step was performed after the automated synthesis by incubating the resin for 10 min in 10% acetic anhydride. The peptides were cleaved from the resin with a cocktail composed of 94:1:2.5:2.5 (*v*/*v*/*v*/*v*) TFA/TIS/EDT/water. After 2 h, the peptides were precipitated from the solution with cold diethyl ether and then lyophilized. The ZnT8N peptide was purified by reversed-phase high-performance liquid chromatography (RP-HPLC; Waters Sp. Warszawa, Poland) on a Ascentis RP-Amide (Supelco—Sigma-Aldrich) and the ZnT8C peptide was purified using RP-HPLC (Knauer Polska, Warszawa, Poland) on a C18 Eurospher II column (Knauer). The eluting solvent A was 0.1% TFA in water, and solvent B was 0.1% TFA in 90% acetonitrile. The high purity of both peptides was confirmed by electrospray ionization mass spectrometry (ESI-MS; Premier, Waters) and potentiometry.

### 4.14. Potentiometry

Potentiometric titrations were performed on a 907 Titrando Automatic Titrator (Metrohm, Herisau, Switzerland) with a Biotrode combined glass electrode (Metrohm). The electrode was calibrated daily by titrating an argon-bubbled 4 mM HNO_3_/96 mM KNO_3_ solution with 0.1 M NaOH. The solubility of the peptide in the HNO_3_/KNO_3_ solution was verified using UV/Vis spectroscopy. Pure peptide, stored anaerobically, was dissolved in 7.5 mL of 0.4 mM HNO_3_/99.6 mM KNO_3_ solution to a final concentration of 180 μM and incubated with 0–0.9 molar equivalents of Zn^2+^ (ZnCl_2_) at 25 °C for 5 min before potentiometric titrations with 0.1 M NaOH. All experiments were performed under argon at 25 °C. The SUPERQUAD and HYPERQUAD programs [48] were used to analyze the data and generate the species distribution diagrams. The formation of complexes was characterized by the general equilibrium process:

(1) $$pM+qH+rL\;\overset{\beta MpHqLr}{\to }\;MpHqLr$$

(2) $$\beta MpHqLr\;=\frac{\left[MpHqLr\right]}{\left[M\right]p\left[H\right]q\left[L\right]r}$$

where *M* represents metal, i.e., Zn^2+^, *L* represents deprotonated ligand, i.e., the 11-residue ZnT8 C-terminal peptide, *H* represents protons and *β* is the stability constant of a complex.

## 5. Conclusions

We demonstrate that the cytosolic CTD of ZnT8 binds nickel ions with a stoichiometry similar to zinc ion binding. Titration of the protein with nickel ions in the presence and absence of cysteine modification and spectroscopic features indicate binding to sulfur in coordination environments that differ significantly in the two variants, revealing for the first time structural differences between the two variants and sulfur coordination in zinc transporter biochemistry, possibly also involving redox biology in human ZnT zinc transporters. We had shown previously that the W/R mutation in the CTD affecting diabetes risk influences metal affinity and elicits differences in physical properties of the protein [21]. How the variants alter the risk of developing diabetes remains an important issue. Our investigations show that this question is related to the specificity and mode of metal binding in the C-terminal domain. Since the only cysteines present in the CTD are the ones in the C-terminus, the participation of these residues, which are typical for the vesicular ZnT proteins, in metal binding is demonstrated as now supported by cryo-EM investigations and high affinity binding of zinc in the absence of added zinc [20]. The metal binding with the participation of cysteine ligands is important for other vesicular transporters (ZnT2, ZnT3 and ZnT4) involved in a wide variety of physiological processes and suggests that other thiophilic metal ions such as cadmium could interfere with the physiological processes in which these transporters are involved. The cryo-EM structure of human ZnT8 demonstrates that the N-terminus connects the metal sites with transmembrane helix 1 (TM1), which together with TM2,4 & 5 is important for the dynamics of the protein in transport [20]. Therefore, there is a conduit that transmits metal binding in the CTD—and the differential effects of the two different amino acids in the variants, which we show affect metal binding to the zinc transporting function of the protein. The N-terminal tail and the type of metal ions bound and the occupancy of the metal binding sites in the CTD are all critical aspects of the functions of the vesicular ZnT transporters.

## Figures and Tables

**Table 1:** Metal analysis (inductively coupled plasma-mass spectrometry (ICP-MS) of zinc and nickel content of 2 μM monomeric ZnT8cR after gel filtration following incubation with 0–10 molar equivalents of Zn^2+^ (n = 3).

Mol. Equiv. Zn^2+^ Added	ZnT8cR			Metal:Monomer
	Zn (μM)	Ni (μM)	Total Metal (μM)	
0	0.03 ± 0.02	0.26 ± 0.03	0.29	0.15
1	2.80 ± 0.07	0.28 ± 0.01	3.03	1.52
2	5.28 ± 0.39	0.24 ± 0.02	5.52	2.76
4	6.65 ± 0.09	0.12 ± 0.01	6.77	3.39
10	5.99 ± 0.30	0.01 ± 0.01	6.00	3.00

**Table 2:** Metal analysis (ICP-MS) of zinc and nickel content of 2 μM monomeric ZnT8cW after gel filtration following incubation with 0–10 molar equivalents of Zn^2+^ (n = 3).

Mol. Equiv. Zn^2+^ Added	ZnT8cW			Metal:Monomer
	Zn (μM)	Ni (μM)	Total Metal (μM)	
0	0.04 ± 0.02	0.33 ± 0.01	0.37	0.19
1	2.48 ± 0.15	0.28 ± 0.02	2.76	1.38
2	5.06 ± 0.21	0.24 ± 0.02	5.30	2.65
4	6.51 ± 0.38	0.12 ± 0.01	6.63	3.32
10	6.23 ± 0.25	0.08 ± 0.01	6.31	3.16

**Table 3:** Metal stoichiometries of ZnT8 variants from total reflection X-ray fluorescence (TXRF and ICP-MS metal analyses following new buffer preparation protocol.

	TXRF		ICP-MS	
	Ni:Monomer	Zn:Monomer	Ni:Monomer	Zn:Monomer
ZnT8cW	0.2 ± 0.1	0.1 ± 0.1	0.5 ± 0.05	0.1 ± 0.06
ZnT8cR	0.3 ± 0.1	0.2 ± 0.1	0.6 ± 0.10	0.2 ± 0.10

**Table 4:** Assaying the sulfhydryls in ZnT8c variants with 5,5′-dithio-bis-(2-nitrobenzoic acid) (DTNB). (A) Free sulfhydryls (thiols) per ZnT8c monomers; (B) Free sulfhydryls (thiols) per ZnT8c monomers after incubating proteins with 15 mM iodoacetamide; (C) Free sulfhydryls (thiols) per ZnT8c monomers after incubating proteins with 15 mM hydrogen peroxide. The experiments were performed in triplicate.

	Variant	A_412_	TNB, µM	ZnT8c, µM	Free Thiol/Monomer
A	ZnT8cR	0.989 ± 0.01	5.59 ± 0.01	2 ± 0.001	2.79 ± 0.02
	ZnT8cW	0.960 ± 0.01	5.42 ± 0.01	2 ± 0.001	2.71 ± 0.01
B	ZnT8cR	0.500 ± 0.005	0.62 ± 0.02	2 ± 0.005	0.31 ± 0.02
	ZnT8cW	0.512 ± 0.002	0.63 ± 0.01	2 ± 0.002	0.31 ± 0.04
C	ZnT8cR	0.450 ± 0.003	0.550 ± 0.005	2 ± 0.005	0.27 ± 0.04
	ZnT8cW	0.420 ± 0.002	0.520 ± 0.008	2 ± 0.003	0.26 ± 0.05

TNB: 2-nitro-5-thiobenzoate.

**Table 5:** Metal stoichiometries of ZnT8c variants from Ni(II) binding data.

	Proteins as Isolated (ICP-MS)	Proteins with Free Cysteines (ICP-MS)	Proteins with Blocked Cysteines (ICP-MS)	Proteins with Free Cysteines (Competition Assay)	Proteins with Blocked Cysteines (Competition Assay)
	Nickel:Monomer	Nickel:Monomer	Nickel:Monomer	Nickel:Monomer	Nickel:Monomer
ZnT8cW	0.50 ± 0.05	2.00 ± 0.01	1.26 ± 0.05	1.98 ± 0.02	1.25 ± 0.07
ZnT8cR	0.60 ± 0.01	1.97 ± 0.01	0.97 ± 0.03	2.00 ± 0.03	1.00 ± 0.02

**Table 6:** Protonation constants (log β) for the ZnT8 8-residue N-terminal peptide and stability constants (log β) for its zinc(II)-peptide complexes, ionic strength 0.1 M (KNO_3_), 25 °C. The protonated residues in each species are predicted based on amino acid p*K*_a_ values.

(Zn*p*)L*q*H*r*	log β	log *K*	Predicted Protonated Residue
LH	10.58 ± 0.02	10.58	Tyrosine side chain
LH_2_	19.84 ± 0.02	9.27	Cysteine sulfhydryl
LH_3_	26.89 ± 0.02	7.05	Histidine side chain
LH_4_	32.71 ± 0.02	5.82	Histidine side chain
ZnLH	18.85 ± 0.02		
ZnL	9.58 ± 0.02		

**Table 7:** Protonation constants (log β) for the ZnT8 11-residue C-terminal peptide, ionic strength 0.1 M (KNO_3_), 25 °C. The protonated residues in each species are predicted based on amino acid p*K*_a_ values.

L*q*H*r*	log β	log *K*	Predicted Protonated Residue
LH	11.29	11.29	Proline α-amino
LH_2_	21.43	10.14	Cysteine sulfhydryl
LH_3_	31.14	9.71	Cysteine sulfhydryl
LH_4_	40.05	8.91	Cysteine sulfhydryl
LH_5_	45.79	5.74	Glutamic acid side chain
LH_6_	50.57	4.78	Aspartic acid side chain
LH_7_	55.33	4.76	Aspartic acid side chain
LH_8_	59.22	3.89	Aspartic acid side chain
LH_9_	63.14	3.92	Aspartic acid α-COOH

**Table 8:** Stability constants (log β) for zinc(II)-peptide complexes of the ZnT8 11-residue C-terminal peptide, ionic strength 0.1 M (KNO_3_), 25 °C.

Zn*p*L*q*H*r*	log β	log *K*
ZnL	15.98	15.98
ZnLH	25.88	9.9
ZnLH_2_	33.14	7.26
ZnL_2_	23.33	7.35
ZnL_2_H	33.85	10.52
ZnL_2_H_2_	44.20	10.35
ZnL_2_H_3_	53.43	9.23
ZnL_2_H_4_	63.01	9.58

## Data Availability

All data are available upon request from the authors.

## Abbreviations

ε	extinction coefficient;
λmax	wavelength of maximal emission;
CD	circular dichroism;
cDNA	complementary DNA;
CTD	C-terminal domain;
CDF	cation diffusion facilitator;
TMD	transmembrane domain;
DTNB	5,5′-dithio-bis-(2-nitrobenzoic acid);
DTT	dithiothreitol;
HEPES	4-(2-hydroxyethyl)-1-piperazineethanesulfonic acid;
SDS	sodium dodecyl sulfate;
ICP-MS	inductively coupled plasma-mass spectrometry;
PAGE	polyacrylamide gel electrophoresis;
TCEP	tris(2-carboxyethyl)phosphine hydrochloride;
TNB	2-nitro-5-thiobenzoate;
ZnT8	member 8 of the ZnT family (*SLC30A8*);
ZnT8c	C-terminal domain of ZnT8 (residues 267–369);
ZnT8cR	C-terminal domain of ZnT8 expressing arginine at position 325, R325 ZnT8;
ZnT8cW	C-terminal domain of ZnT8 expressing tryptophan at position 325, W325 ZnT8;
TXRF	total reflection X-ray fluorescence
